# Utility of the Post-Reflux Swallow-Induced Peristaltic Wave Index and Mean Nocturnal Baseline Impedance for the Diagnosis of Gastroesophageal Reflux Disease Phenotypes in Children

**DOI:** 10.3390/children11070773

**Published:** 2024-06-26

**Authors:** Radu Samuel Pop, Daniela Pop, Lăcrămioara Eliza Chiperi, Vlad-Ionuț Nechita, Sorin Claudiu Man, Dan Lucian Dumitrașcu

**Affiliations:** 1 3rd Department of Pediatrics, “Iuliu Hațieganu” University of Medicine and Pharmacy, 400217 Cluj-Napoca, Romania; pop.daniela@umfcluj.ro (D.P.); claudiu.man@umfcluj.ro (S.C.M.); 2 3rd Pediatric Clinic, Clinical Emergency Hospital for Children, 400217 Cluj-Napoca, Romania; 3 Department of Pediatrics, George Emil Palade University of Medicine, Pharmacy, Sciences and Technology, 540136 Târgu Mureș, Romania; lacramioara-eliza.pop@umfst.ro; 4 Department of Medical Informatics and Biostatistics, “Iuliu Hațieganu” University of Medicine and Pharmacy, 400349 Cluj-Napoca, Romania; nechita.vlad@umfcluj.ro; 5 2nd Department of Internal Medicine, “Iuliu Hațieganu” University of Medicine and Pharmacy, 400006 Cluj-Napoca, Romania; ddumitrascu@umfcluj.ro

**Keywords:** GERD, MNBI, PSPW, children

## Abstract

(1) Objectives: Assessment of novel impedance parameters such as the post-reflux swallow-induced peristaltic wave (PSPW) index and mean nocturnal baseline impedance (MNBI) have been proposed to enhance the accuracy of gastroesophageal reflux disease (GERD) diagnosis. We aimed to evaluate the clinical value of MNBI and the PSPW index in discerning different phenotypes of GERD in children. (2) Methods: We conducted a prospective, observational study that included 49 children aged 5–18 years, referred for MII-pH monitoring due to negative endoscopy and persisting gastroesophageal reflux symptoms despite acid-suppressant treatment. The PSPW index and MNBI were assessed along with conventional metrics. (3) Results: Using a receiver operating characteristic (ROC) curve analysis, MNBI (AUC 0.864) and the PSPW index (AUC 0.83) had very good performance in differentiating between non-erosive reflux disease (NERD) and functional phenotypes. The PSPW index (AUC 0.87) discriminated better between functional heartburn (FH) and reflux hypersensitivity (RH) compared to the MNBI (AUC 0.712). A PSPW cut-off value of 65% provided a sensitivity of 76.9% and a specificity of 90% in distinguishing FH and RH. The PSPW index (AUC 0.87) proved to have better performance than the MNBI (AUC 0.802) in differentiating between FH and non-FH patients. MNBI diagnosed FH with a sensitivity of 84% and a specificity of 80.6% at a cut-off value of 2563 Ω. (4) Conclusions: The PSPW index and MNBI are useful to distinguish between GERD phenotypes in pediatric patients.

## 1. Introduction

Gastroesophageal reflux disease (GERD) is defined as the reflux of gastric contents into the esophagus, leading to a spectrum of bothersome symptoms and potential complications [1,2,3]. 

The prevalence of pediatric GERD differs among various age groups and populations, with a global range of 0.2% to 40% [4]. In Asian infants, the prevalence was found to peak at 26.5% at six weeks of life and decreased to 1.1% by 12 months of age [5]. In the United States, a range of 5.2% to 8.2% of children between the ages of 10 and 17 experience symptoms of GERD on a weekly basis [6]. Also, 12.4% of children who underwent endoscopy were diagnosed with erosive esophagitis [7]. Conversely, the United Kingdom had a reported rate of 10.9 cases per 1000 inhabitants per year from 2000 to 2005, when individuals were newly diagnosed with reflux esophagitis [8]. In France, about 10% of children and adolescents show gastroesophageal reflux (GER) symptoms and about 6% have GERD [9]. A systematic review noted that in children older than 18 months, GERD symptoms are present in >10% on a weekly basis and in 25% on a monthly basis, with an overall pooled prevalence from four cross-sectional studies of 26.9% [4]. The prevalence rate of endoscopically proven reflux esophagitis in children in Korea was reported to be 28.7%, according to Yang et al. [10].

In pediatric patients, GERD can present with a variety of manifestations. Symptoms observed in infants and young children include regurgitation, gagging, irritability, crying episodes, feeding difficulties, failure to thrive, and sleep disturbances. Additionally, infants may exhibit extraesophageal symptoms such as coughing, wheezing, arching of the back and neck, and even life-threatening events like choking or apnea. In older children and adolescents, clinical presentations resemble those seen in adults, including gastrointestinal symptoms like heartburn, retrosternal or epigastric pain, dysphagia, chronic regurgitation, nausea, nocturnal pain, and sour burps or extraesophageal manifestations, including chronic cough, wheezing, hoarseness, sore throat, and dental erosions [2,6,11,12]. 

Visible lesions of the esophageal mucosa detected during endoscopy serve as diagnostic markers for erosive reflux disease (ERD) [2,8,10,13]. However, over time, a subset of patients with typical reflux symptoms showed a poor response to acid suppression, indicating that acid might not be the only cause behind their symptoms [14]. Persistent symptoms despite acid suppression prompted further diagnostic investigations, like combined 24 h multichannel intraluminal impedance and pH monitoring (MII-pH), revealing the involvement of other factors such as weakly acidic reflux, esophageal hypersensitivity, functional issues in symptom perception, and motility disorders [15,16,17,18,19,20,21]. 

MII-pH monitoring is capable of detecting both acid and non-acid reflux by monitoring changes in electrical impedance during the passage of liquid, solid, or gas bolus between measuring electrodes at various esophageal levels, irrespective of the physical or chemical characteristics of the bolus, as evidenced by several studies [22,23,24,25,26,27,28,29,30,31,32,33]. MII-pH monitoring is the most sensitive diagnostic tool for assessing GERD, describing the correlation between reflux and symptoms in patients exhibiting typical and atypical symptoms [12,34,35,36,37,38,39,40,41,42] The current European and North American guidelines [2] recommended 24 h MII-pH monitoring in children whose endoscopy results are normal but continue to have symptoms despite receiving acid suppression therapy for 4–8 weeks.

The contemporary understanding of GERD in both adults and children now portrays it as a spectrum of phenotypes [43,44] rather than a singular diagnosis, as emphasized in the 2016 Rome IV classification of functional esophageal disorders [2]. Patients with negative endoscopy, having abnormal esophageal acid exposure observed during combined 24 h MII-pH, represent the phenotype that is described as non-erosive reflux disease (NERD) [23,45,46,47,48,49,50,51]. Those patients with normal esophageal acid exposure and positive symptom correlation are diagnosed with reflux hypersensitivity (RH), and those with normal esophageal acid exposure and negative symptom correlation are categorized as having functional heartburn (FH) [43]. Blasi et al. [52] described a fourth phenotype, normal reflux index not otherwise specified (“normal RI-NOS”), consisting of patients characterized by normal esophageal acid exposure time and an unreliable symptom association due to less than three reported symptoms during MII-pH monitoring. While not explicitly delineated by the Rome IV criteria, these patients are unlikely to have a positive response to acid-suppressive medication because their esophageal acid exposure is normal.

Proper classification into these subgroups holds significant therapeutic implications, as patients in each category may exhibit differential responses to medical and surgical interventions. There are little data regarding the prevalence of GERD phenotypes in pediatric patients and the treatment outcome [52,53].

Two new impedance-pH metrics have been proposed recently: post-reflux swallow-induced peristaltic wave (PSPW) index and mean nocturnal baseline impedance (MNBI) [54]. MNBI quantifies the baseline electrical resistance of the esophageal mucosa during intervals with no reflux episodes, potentially indicating mucosal integrity and the burden of reflux [55,56]. Conversely, the PSPW, characterized by an antegrade esophageal contraction subsequent to reflux episodes, mirrors the efficacy of esophageal peristalsis in the reflux clearance from the esophageal lumen [57]. 

The MNBI and PSPW index have emerged as promising objective metrics for improving the diagnostic accuracy of MII-pH monitoring, especially in distinguishing between various phenotypes of GERD [58,59]. Very little data have been published about the utility of the MNBI and PSPW index in children.

In adult studies, these parameters are regarded as providing a more accurate delineation of the GERD phenotype, especially in cases of inconclusive acid exposure time [58,60], enhancing the diagnostic process [57,61,62] and improving predictions of the efficacy of anti-reflux therapies [63,64,65]. The MNBI and PSPW index have demonstrated a high diagnostic yield for delineating GERD phenotypes of GERD, with several authors [54,59,66,67,68] advocating their routine assessment despite concerns about the fact that the calculation is time-consuming and can vary due to manual measurement. In this regard, expert recommendations for a standardized identification of the PSPW index in clinical practice were provided by the recent Wingate Consensus [69].

The addition of the PSPW index and MNBI to MII-pH monitoring protocols in adults has notably enhanced GERD diagnostic abilities, offering clinicians essential ways to improve patient care and treatment outcomes [70,71].

Nonetheless, the utility of the PSPW index and MNBI in pediatric GERD remains not fully comprehended. While research conducted on adult cohorts has demonstrated the diagnostic potential, the distinctive challenges and factors inherent to the pediatric population may impact the relevance and interpretation of these parameters [72]. 

The aim of this study was to evaluate the clinical value of the MNBI and PSPW index, to explore the potential association between these parameters, and to assess their effectiveness in discerning different phenotypes of GERD in children.

## 2. Materials and Methods

### 2.1. Study Design and Patient Selection

We conducted a prospective, observational, single-center study between October 2019 and December 2023 at Emergency Clinical Hospital for Children, 3rd Pediatric Clinic, that included pediatric patients aged 5–18 years, referred for MII-pH monitoring due to normal endoscopy and persisting gastroesophageal reflux symptoms despite acid-suppressing treatment. We included patients who were able to describe typical GERD symptoms like heartburn, epigastric pain, and regurgitation. Other inclusion criteria were the absence of esophageal erosions during endoscopy and persisting symptoms after at least 4 weeks of acid-suppressing treatment. Only recordings with a duration of at least 20 h were included. The exclusion criteria comprised individuals presenting with erosive or eosinophilic esophagitis, a history of gastric or esophageal surgical interventions, diagnosed esophageal motor dysfunctions, peripheral and autonomic neuropathies, those with neurological or psychiatric impairments, and those failing to meet the predefined inclusion criteria. We used a convenience sample because the proportion of GERD patients is not known among the Romanian pediatric population.

This study was conducted in accordance with the Declaration of Helsinki and was approved by the local ethics committee (Nr. 299/11.09.2019). Written informed consent was also obtained from parents of the children before inclusion in the study. 

### 2.2. Twenty-Four-Hour Impedance-pH Monitoring

Twenty-four-hour MII-pH (Sandhill Scientific, Highlands Ranch, CO, USA) monitoring was conducted concurrently in all patients. Catheters with a diameter of 2.13 mm/6.4 Fr with seven impedance sensors and one pH sensor were used. Prior to each examination, catheters were pre-calibrated in buffer solutions with pH of 4 and 7 and after that, were inserted via the anterior nares. The six impedance sensors are located at 3, 5, 7, 9, 15, and 17 cm above the lower esophageal sphincter at Z6 = 3 cm, Z5 = 5 cm, Z4 = 7 cm, Z3 = 9 cm, Z2 = 15 cm, and Z1 = 17 cm, respectively. The catheter placement was established according to the formula proposed by Mutalib et al. [73] and checked through fluoroscopy. Patients had fasted for at least 4 h prior to the catheter insertion. Proton pump inhibitors, histamine H2-receptor blockers, and prokinetic drugs were stopped seven days prior to the investigation in accordance with the current recommendation [74]. 

Liquid reflux was defined as a drop in impedance to >50% of the baseline impedance (BI) value starting from the most distal sensors and propagating up to at least the next two proximal sensors. Reflux was classified as acid at pH < 4 or non-acid at pH > 4 for the purpose of the study. An abnormal number of reflux episodes was considered >70 in 24 h, although a rationale for these cut-off values does not exist yet. A reflux index (RI), also known as acid exposure time (AET), above 7% was considered abnormal; a reflux index below 3% was normal, and a reflux index between 3 and 7% was indeterminate, according to most of the guidelines [1,41,74]. Classic parameters were calculated automatically by the Sandhill Bioview Analysis software: mean acid clearance time (MACT); mean bolus clearance time (MBCT); symptom index (SI) representing the number of reflux-related symptoms in the total number of symptom episodes (×100%), considered to be positive when >50%; symptom sensitivity index (SSI, reported as %), the percentage of symptom-associated GER events divided by the total number of GER events, with a value of >10% considered significant; and the symptom association probability (SAP), which represents the likelihood that the patient’s symptoms are related to reflux episodes. By agreement, the SAP is considered to be positive when it is >95% [2]. We considered a reliable SAP when the symptom was reported at least 3 times. In patients with indeterminate RI and positive SAP and/or SI, pathological number of reflux episodes distinguished between NERD patients and those with RH.

MNBI was measured using the two most distal impedance channels (MNBI at 3 cm above the low esophageal sphincter (LES) and MNBI at 5 cm above the LES) by two methods. The conventional MNBI was measured as described by Martinucci et al. [75] during the nighttime while the patients were in a recumbent position. MNBI value was determined from the average value of three stable 10 min intervals at 1:00 AM, 2:00 AM, and 3:00 AM, excluding pH drops, swallows, or reflux episodes.

PSPW is defined by a 50% drop in impedance occurring within 30 s of a reflux episode, starting at the most proximal impedance channel and extending to the most distal one, followed by at least a 50% return to baseline [57]. Manual analysis of the number of episodes followed by the swallow peristaltic wave was required as the software does not have an automatic measuring function. The PSPW index was calculated by dividing the number of PSPWs by the number of reflux events. Time periods, including swallows, refluxes, and pH drops, were avoided.

### 2.3. Statistical Methods

For the statistical analysis, Statistical Package for Social Sciences (IBM SPSS^®^ version 29.0.2.0 for Windows, IBM Crop. Armonk, New York, NY, USA) and R version 4.0.5 with R Commander were used (R Foundation for Statistical Computing, Vienna, Austria). Shapiro–Wilk test, skewness, and kurtosis were used to evaluate quantitative data distribution. Quantitative data with normal distribution were presented as mean ± standard deviation or median and interquartile ranges for non-normal distribution. We used the Chi-square test to compare frequencies and Fisher’s exact test when the expected frequencies were under 5. ANOVA analysis was used when comparing multiple groups, and Student’s test was used when we compared quantitative data. To evaluate the correlation between quantitative data, we used Pearson’s correlation coefficient. The diagnostic performance of parameters such as MNBI and PSPW was evaluated using the area under the ROC curve (AUROC) analysis, using the maximum Youden index. Cut-off values were determined based on the ROC curve to optimize sensitivity and specificity, ensuring the best overall diagnostic accuracy. A *p*-value of less than 0.05 was considered statistically significant. 

## 3. Results

Fifty-one patients were eligible for our study. Two were excluded due to technical issues during the recording. Of the forty-nine patients included for analysis, 12 (24.5%) had non-erosive reflux disease (NERD), 20 (40.8%) had reflux hypersensitivity (RH), 13 (26.5%) had functional heartburn, and 4 (8.2%) had a normal reflux index not otherwise specified (RI-NOS). The general characteristics of the patients are presented in Table 1. Our study included 23 male patients (46.9.6%) and 26 female patients (53.1%). There were significantly more male patients having NERD and normal RI-NOS. No difference was observed regarding the BMI percentile among the phenotypes.

**Figure 1:**
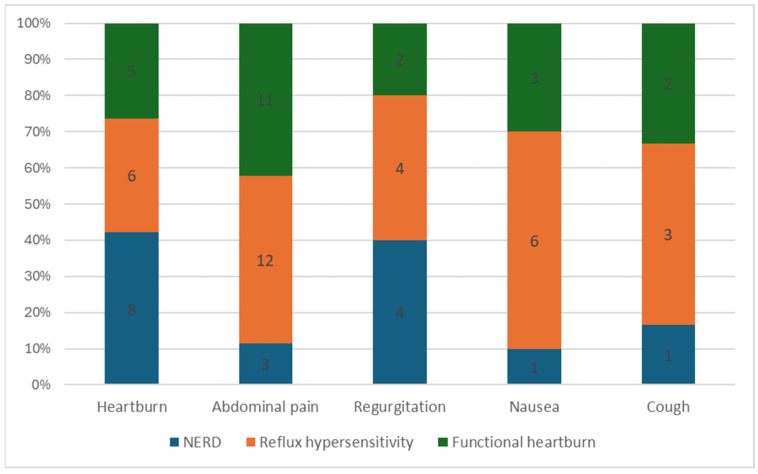
Symptoms reported during MII-pH monitoring by phenotype.

Figure 1 illustrates the prevalence of frequently reported symptoms during MII-pH monitoring in the study population, as well as their distribution among NERD, RH, and FH patients. Other reported symptoms included globus, belching, and hiccups. There was no statistically significant association found between symptoms and gender.

The MII-pH conventional metrics and the novel parameters among different phenotypes are shown in Table 2. NERD patients had a significantly higher number of reflux episodes compared to non-NERD patients (*p* = 0.003). There was no significant difference in bolus clearance time among the four groups. FH and normal RI-NOS patients had fewer proximal reflux episodes compared to NERD patients.

**Figure 2:**
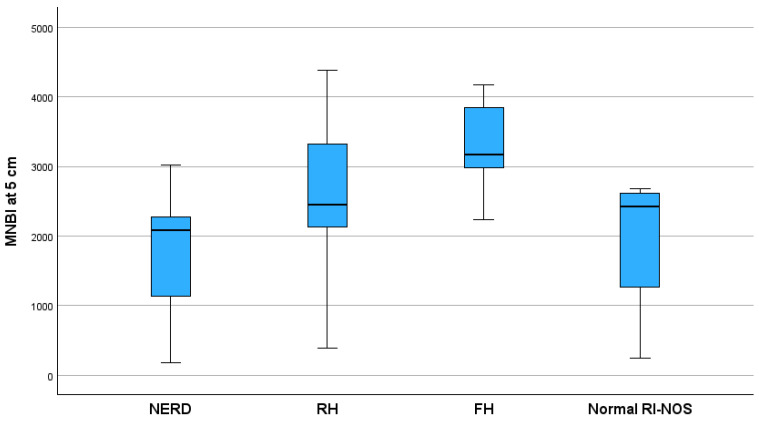
Distribution of MNBI at 5 cm above the LES values between GERD phenotypes (*p* = 0.004). MNBI, mean nocturnal baseline impedance; NERD, non-erosive reflux disease; RH, reflux hypersensitivity; FH, functional heartburn; Normal RI-NOS, normal reflux index not otherwise specified.

**Figure 3:**
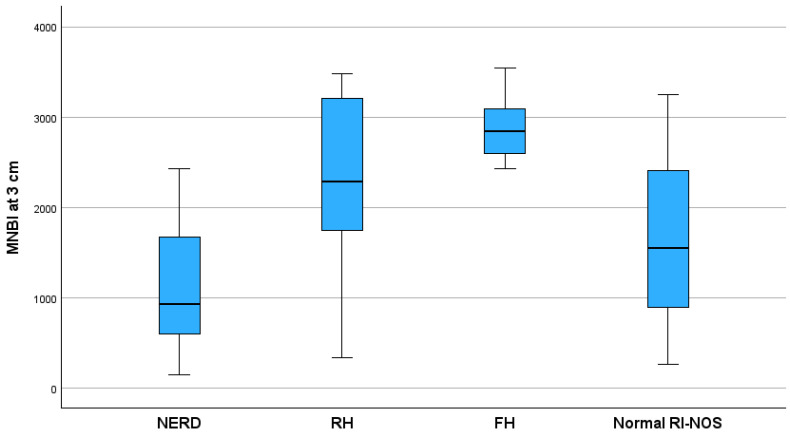
Distribution of MNBI at 3 cm above the LES values between GERD phenotypes (*p* < 0.001). MNBI, mean nocturnal baseline impedance; NERD, non-erosive reflux disease; RH, reflux hypersensitivity; FH, functional heartburn; Normal RI-NOS, normal reflux index not otherwise specified; LES, low esophageal sphincter.

A significant negative correlation was found between the MNBI at 3 cm and the reflux index (r = −0.52, *p* < 0.001). We found a significant statistical difference between the MNBI at 5 cm and the MNBI at 3 cm values among GERD phenotypes (*p* = 0.001) presented in Figure 2 and Figure 3 FH patients had the highest value of MNBI at 5 cm, while NERD patients had the lowest value, significantly different from patients with normal acid exposure (*p* < 0.001). There was no difference between the MNBI at 3 cm, MNBI at 5 cm, and PSPW index values comparing FH patients with those categorized as normal RI-NOS.

**Figure 4:**
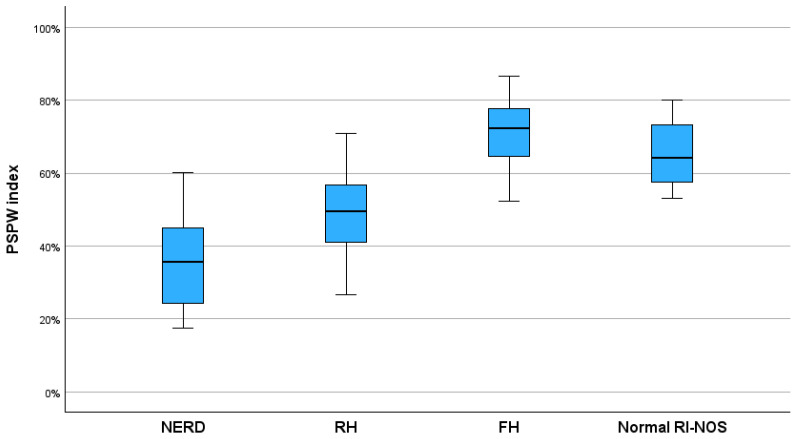
Distribution of PSPW index values among the GERD phenotypes. PSPW, post-reflux swallow peristaltic wave; NERD, non-erosive reflux disease; RH, reflux hypersensitivity; FH, functional heartburn; Normal RI-NOS, normal reflux index not otherwise specified.

The PSPW index values across GERD phenotypes were significantly different (Figure 4). Patients with NERD had the lowest PSPW values, followed by the RH and FH patients. We found a significantly higher PSPW index among patients with FH compared to those with RH (*p* < 0.01) and compared to NERD patients (*p* < 0.01). 

We assessed the performance of the MNBI at 5 cm, MNBI at 3 cm, and PSPW index in differentiating between non-erosive phenotypes (Table 3). 

**Figure 5:**
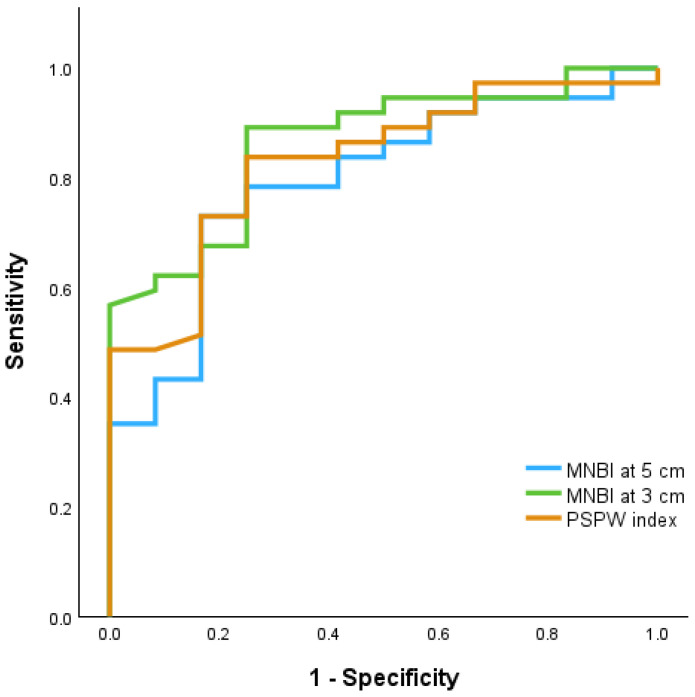
ROC curves for MNBI at 5 cm, MNBI at 3 cm, and PSPW index to differentiate NERD patients from non-NERD patients.

A receiver operating characteristic (ROC) curve analysis revealed that the MNBI at 3 cm (AUC 0.864 and 95% CI 0.756–0.972) and the PSPW index (AUC 0.83 and 95% CI 0.707–0.953) demonstrated excellent ability to differentiate between NERD and non-NERD phenotypes (Figure 5).

The MNBI at 3 cm was able to distinguish NERD from non-NERD phenotypes with a sensitivity of 89.19% and a specificity of 75% at a cut-off value of 1457 Ω. 

In distinguishing between FH and RH patients, the PSPW exhibited greater effectiveness, having an AUROC of 0.87 (95% CI 0.734–1.008) compared to the MNBI at 5 cm with an AUC of 0.712 (CI 95% 0.532–0.891). A PSPW cut-off value of 65% provided a sensitivity of 76.9% and a specificity of 90% in discriminating RH from FH. The MNBI showed good performance, with a sensitivity of 92.32% and a specificity of 55% at a cut-off value of 2537 Ω.

All three metrics showed an excellent ability to discriminate functional heartburn FH from the rest of the phenotypes. The PSPW index (AUC 0.87 and CI 95% 0.103–0.386) had a similar performance to the MNBI at 3 cm (AUC 0.802 and CI 95% 0.676–0.928), being able to diagnose FH with a sensitivity of 76.9% and a specificity of 88.9% at a cut-off value of 64%. However, the MNBI at 3 cm had the capacity of predicting the presence of FH with a sensitivity of 84% and a specificity of 80.6% at a cut-off value of 2563 Ω.

For patients with inconclusive reflux index (3–7%), an acceptable differentiation capacity between NERD and RH was observed for the MNBI at 5 cm, with a sensitivity of 66.6% and a specificity of 66.67% (AUC = 55.6%) at a cut-off value of 2262 Ω.

A strong correlation was found between the MNBI at 3 cm and reflux index (r = −0.52 and *p* < 0.001). We evaluated if there was any correlation between the PSPW index and the MNBI at 5 cm and the MNBI at 3 cm. Using the Pearson correlation coefficient, we observed a significant statistically positive correlation between MNBI and PSPW (r = 0.438 and *p* = 0.002).

## 4. Discussion

Due to the wide use of MII-pH monitoring in patients with no esophageal lesions detected during upper endoscopy, GERD has evolved to a spectrum of phenotypes, each characterized by its distinct underlying pathophysiological mechanisms. Understanding the mechanisms behind the range of GERD phenotypes in children is essential for tailoring effective treatment. The prevalence of non-erosive phenotypes was examined in two studies using the Rome IV criteria among children ≥5 years of age with negative endoscopy and off-therapy. Mahoney et al. [53] showed that among 45 children, 27% were diagnosed as having NERD, 29% as RH, and 44% as FH. Blasi et al. [52] confirmed, in a larger multicenter study, that among 68 children, FH was the most prevalent phenotype at 38.2%, followed by NERD at 26.5% and acid reflux hypersensitivity (RH) at 20.6%. The prevalence of NERD, as shown in our study, is 24.5%, which is consistent with the data published. We found a different prevalence of RH (40.8%) and FH (26.5%). By categorizing the 8.2% of our patients with normal reflux index not otherwise specified (RI-NOS) as functional heartburn, the prevalence of FH would be similar to the data presented in the previous studies. Adult studies applying the same criteria found a lower prevalence of RH (14.6–35%); FH ranged from 21.9 to 35.35%, and there was a higher prevalence of NERD (40–58.5%) [77,78,79]. 

### 4.1. MNBI

Studies showed that acid exposure disrupts intercellular junctional complexes within the esophageal epithelium. This often causes a leakage between cells and the dilation of intercellular spaces. This damaged epithelium has lower electrical resistance compared to the normal one, resulting in low impedance in pH-MII [80,81,82,83]. We found a significant correlation between the MNBI at 3 cm and acid exposure time (r = −0.52, *p* < 0.001). In our study, a significantly different MNBI at 3 cm was found in children with an abnormal reflux index compared to those with a normal reflux index. Several studies have shown that pediatric patients with abnormal acid exposure have significantly lower MNBI values compared to those with normal acid exposure time. Rosado-Arias et al. [84] and Blasi et al. [52] reported that pediatric patients with severe esophagitis have a lower MNBI than patients with non-severe esophagitis. This suggests that measuring nocturnal baseline impedance may be a valuable diagnostic tool, potentially eliminating the need for upper gastrointestinal endoscopy in assessing esophagitis. Blasi et al. [52] described a lower MNBI among children with NERD compared to those with functional phenotypes of GERD. Sabban et al. [85] reported statistically significantly higher MNBI values in children with FH compared to those diagnosed with RH (*p* = 0.001).

These findings align with data from adult patients, where MNBI has been shown to be lower in patients with RH and NERD compared to those with functional heartburn and healthy individuals [86]. Moreover, MNBI has been reported to differentiate FH patients from those having NERD with increased accuracy [65,87].

Using a ROC curve analysis, we observed an excellent ability of the MNBI at 3 cm (AUC 0.864 and CI 95% 0.756–0.972) in differentiating between NERD and non-NERD phenotypes.

The MNBI at 3 cm was able to distinguish NERD from non-NERD phenotypes with a sensitivity of 89.19% and a specificity of 75% at a cut-off value of 1457 Ω. To the best of our knowledge, there is no published data on the accuracy of MNBI in distinguishing between NERD pediatric patients and those with RH or FH. There is also limited data on the role of MNBI in distinguishing GERD in pediatric patients. Rosado-Arias et al. [88] showed that MNBI at channel 6 had an acceptable capacity to diagnose GERD in children, with an AUC of 0.668, a sensitivity of 100%, and a specificity of 45% at a cut-off value of 2183 Ω. Eiamkulbutr et al. [72] showed that at a cut-off value of 1466 ohms, with a sensitivity of 50.0% and a specificity of 33.33%, MNBI could diagnose GERD in children. Concerning the overall ability to diagnose GERD, Frazzoni et al. [57] found an AUC for the MNBI of 0.876 (95% confidence interval, 0.833–0.918, and with the best cut-off value of 2292 Ω). Another study [61] reported a cut-off of 2061 ohms, with an AUC of 0.792. This cut-off yielded a sensitivity of 74.9% and a specificity of 67.4% [61]. There is no international consensus on the MNBI or PSPW index cut-off values, neither in children nor in adults. A multicenter study has redefined the cut-off values for the MNBI as 2000 Ω [67]. Another study conducted on the Chinese population indicated that the positive cut-off values for proximal MNBI and distal MNBI were 1960.5 Ω and 1890.6 Ω, respectively, based on an ROC analysis [89]. Additional research and validation are needed to create standardized diagnostic criteria. In distinguishing between FH and RH patients, we found that the MNBI at 5 cm had an AUC of 0.712 (CI 95% 0.532–0.891), showing good performance. It had a sensitivity of 92.32% and a specificity of 55% at a cut-off value of 2537 Ω. Frazzoni et al. [55] reported that the PSPW index had an AUC of 0.924 (95% confidence interval 0.879–0.969) and the MNBI had an AUC of 0.864 (95% confidence interval 0.809–0.919) in distinguishing between FH and RH patients, suggesting that a diagnosis of RH can be confirmed in most of the cases with a combined assessment of the MNBI and PSPW index, independently of classic association metrics like SI or SAP. Gao et al. [90] showed that MNBI yielded areas under the curve of 0.643 (95% CI 0.570–0.716), significantly distinguishing between FH and RH. Impairment of mucosal integrity and chemical clearance can explain the hypersensitivity of the esophageal mucosa even to normal acid exposure. 

Both the MNBI at 5 cm and the MNBI at 3 cm showed an excellent ability to discriminate functional heartburn FH from the rest of the phenotypes. The MNBI at 3 cm (AUC 0.802 and CI 95% 0.676–0.928) was able to diagnose FH with a sensitivity of 76.9% and a specificity of 88.9% at a cut-off value of 64%. However, the MNBI at 3 cm had the capacity of predicting the presence of FH with a sensitivity of 84% and a specificity of 80.6% at a cut-off value of 2563 Ω.

The MNBI at 5 cm demonstrated an acceptable differentiation capacity between NERD and RH for patients with inconclusive reflux index (3–7%), with a sensitivity of 66.6% and a specificity of 66.67% (AUC = 55.6%) at a cut-off value of 2262 Ω. In adult studies, MNBI has proven effective in precisely evaluating GERD phenotypes, particularly in situations where conventional metrics yield unclear outcomes. [16,58,59,65,91].

In pediatric patients, low MNBI values are correlated with endoscopically proven reflux esophagitis. It may become a predictor of esophageal damage, offering the advantage of being less invasive and not requiring fasting or sedation. 

### 4.2. PSPW

The PSPW index values across GERD phenotypes were significantly different. We found a significantly higher PSPW index among patients with FH compared to those with RH (*p* < 0.01) as well as compared to NERD patients (*p* < 0.01). Two studies conducted on pediatric patients [44,92] reported a lower PSPW index in pediatric patients diagnosed with GERD compared to those having NERD or functional phenotypes, demonstrating a negative correlation between the PSPW index and acid exposure time. Blasi et al. [29] confirmed this finding, suggesting a possible connection between a lower PSPW index and increased acid exposure time.

In our study, the PSPW index had an excellent performance (AUC 0.83 and CI 95% 0.707–0.953) with a very good capacity to differentiate between NERD and non-NERD phenotypes. Frazzoni et al. [66] reported an AUC for the PSPW index of 0.886, indicating its high discriminatory ability between NERD and FH in adult patients.

Sabban et al. [85] conducted a study in which patients with RH had significantly lower PSPW index values compared to those with FH (*p* = 0.01). In another study, Sabban et al. [93] found a lower PSPW index in pediatric patients with respiratory symptoms suggesting GERD compared to patients with typical gastrointestinal symptoms. 

Differentiating between RH and FH is crucial due to different approaches in means of treatment, but it remains challenging, especially when conventional metrics are inconclusive [55]. Our study reveals that the PSPW index had an excellent ability to discriminate between RH and FH patients, with an AUC of 0.87 (95% CI 0.734–1.008). At a cut-off value of 54.5%, it provided a sensitivity of 76.9% and a specificity of 90% in discriminating RH from FH. Similar data were obtained by Gao et al. [90] in adult patients, revealing a cut-off value of 0.728 (95% CI 0.661–0.796). 

Frazzoni et al. [94] demonstrated in a prospective study conducted in multiple centers on adults that the PSPW index was the only parameter able to predict the lack of response to GERD treatment (OR 1.082 and *p* = 0.007). 

A recent study among patients exhibiting extraesophageal manifestations of GERD was conducted by Ribolsi et al. [68] and revealed that assessing the PSPW index enhanced the diagnostic accuracy of impedance-pH monitoring when compared to classic metrics like AET, SAP, and the presence of typical symptoms. They reported a sensitivity of 75% and a negative predictive value (NPV) of 76% for GERD diagnosis.

A study conducted on adult patients by de Bortoli et al. [95] revealed a negative strong correlation between the PSPW index and the bolus clearance time (r = −0.889), highlighting the role of esophageal clearance. Furthermore, a positive correlation between the PSPW index and MNBI values has been found (r = 0.722) [58], indicating the role of chemical clearance in preserving mucosal integrity. Rogers et al. [62] showed that normal PSPW is associated with low acid exposure and low PSPW index is associated with esophageal hypomotility.

As research in this area progresses, the role of artificial intelligence (AI) is likely to become increasingly significant in improving the diagnosis and management of GERD. Studies have shown that AI systems can aid in the automated analysis of MII-pH tracings, particularly in calculating MNBI and the PSPW index [96,97]. The accuracy of AI in identifying the PSPW has been reported to be as high as 82% [98]. The integration of AI in the analysis of MII-pH tracings for GERD diagnosis shows promise in enhancing the accuracy and efficiency of measuring MNBI and PSPW index. 

Our study had some limitations. First, the relatively small sample size may have limited the generalizability of our findings. Another limitation was that we included patients from a single tertiary center, which may not be representative of broader populations. We did not perform esophageal manometry to determine the exact location of the LES. This could lead to some uncertainty in our findings regarding the MNBI at different levels. Future studies should include esophageal manometry to improve the accuracy of LES localization. The current MII-pH software does not provide the automatic measurement of the novel metrics, so manual measurements of the MNBI and PSPW index were made, and they are prone to human error.

Future multicenter prospective studies are warranted in order to establish cut-off values and elaborate recommendations for standardized measurements. 

## 5. Conclusions

Our study supports the use of the PSPW index and MNBI to distinguish between GERD phenotypes in pediatric patients. Multicenter large-scale studies are needed to establish an evidence-based recommendation for the assessment of the PSPW index and MNBI as standard measurements of MII-pH monitoring in children with negative upper endoscopy and suggestive GERD symptoms despite treatment.

## Figures and Tables

**Table 1:** Demographic characteristics among phenotypes.

	NERD (n = 12)	RH (n = 20)	FH (n = 13)	Normal RI-NOS (n = 4)	*p*-Value
Age years (mean ± SD)	13.42 ± 2.27	13.4 ± 3.6	14.46 ± 2.07	12.75 ± 4.72	0.69
Sex (male %)	75	40	23.08	75	0.03
BMI for age—percentile (%)	49.9 ± 28.3	47.4 ± 30.7	51.4 ± 38.4	63.5 ± 40.84	0.85

NERD, non-erosive reflux disease; RH, reflux hypersensitivity; FH, functional heartburn; Normal RI-NOS, normal reflux index not otherwise specified; SD, standard deviation; NS, not significant. BMI for age—percentile, body mass index for age—percentile [76].

**Table 2:** Esophageal multichannel intraluminal impedance and pH monitoring parameters.

	NERD	RH	FH	Normal RI-NOS	*p*-Value
Reflux index (mean ± SD)	7.83 ± 1.2	1.53 ± 1.2	0.98 ± 098	0.9 ± 0.61	<0.001
Total reflux episodes (mean ± SD)	94.7 ± 44.3	66.3 ± 45.4	32.8 ± 15	36 ± 29.5	0.003
Acid reflux episodes	76.3 ± 37.8	31.6 ± 22.2	18.9 ± 11.8	20.8 ± 11.4	<0.001
Proximal reflux episodes	53.4 ± 24	34 ± 27.1	15.8 ± 6.7	17.3 ± 15.02	0.001
Mean acid clearance time (s) (mean ± SD)	120.6 ± 79.8	62.7 ± 46.7	53.8 ± 51.8	44.5 ± 30.3	0.01
Bolus clearance time (s) (mean ± SD)	12.3 ± 3.55	15.4 ± 5.9	12.5 ± 3.18	16.5 ± 6.76	0.16
MNBI at 5 cm above LES	1812 ± 875	2645 ± 984	3256 ± 613	1941 ± 1146	0.001
MNBI at 3 cm above LES	1121 ± 773	2281 ± 890	2882 ± 526	1652 ± 1225	<0.001
PSPW index (%)	36.1 ± 13.9	47.6 ± 14.8	69.9 ± 14.4	65.4 ± 11.3	<0.001

NERD, non-erosive reflux disease; RH, reflux hypersensitivity; FH, functional heartburn; Normal RI-NOS, normal reflux index not otherwise specified; SD, standard deviation; NS, not significant; MNBI, mean nocturnal baseline impedance; PSPW, post-reflux swallow peristaltic wave; LES, low esophageal sphincter.

**Table 3:** Performance of MNBI at 5 cm, MNBI at 3 cm, and PSPW index in delineating GERD phenotypes.

NERD vs. Non-NERD Phenotypes (RH, FH, and Normal RI-NOS)							
	**AUC (CI 95%)**	**Cut-off**	**Sensitivity**	**Specificity**	**PPV**	**NPV**	***p*-Value**
MNBI at 5 cm	0.795 (0.656–0.934)	2328 Ω	72.97%	83.33%	93.1%	50%	<0.001
MNBI at 3 cm	0.864 (0.756–0.972)	1457 Ω	89.19%	75%	91.67%	69.23%	<0.001
PSPW index	0.83 (0.707–0.953)	43%	83.78%	75%	91.18%	60%	<0.001
**FH vs. RH**							
	AUC (CI 95%)	Cut-off	Sensitivity	Specificity	PPV	NPV	*p*-Value
MNBI at 5 cm	0.712 (0.532–0.891)	2537 Ω	92.32%	55%	57.14%	91.67%	<0.001
MNBI at 3 cm	0.70 (0.513–0.887)	2596 Ω	70%	64.71%	64.71%	87.5%	<0.001
PSPW index	0.87 (0.734–1.008)	65%	76.9%	90%	83.3%	85.71%	<0.001
**FH vs. Normal RI-NOS**							
	AUC (CI 95%)	Cut-off	Sensitivity	Specificity	PPV	NPV	*p*-Value
MNBI at 5 cm	0.865 (0.688–1.043)	2979 Ω	76.92%	100%	100%	57.14	<0.001
MNBI at 3 cm	0.788 (0.424–1.153)	1706 Ω	100%	75%	92.86	100%	<0.001
PSPW index	0.625 (0.322–0.928)	70%	61.54%	75%	88.89%	37.5%	<0.001
**FH vs. non-FH (NERD, RH, and Normal RI-NOS)**							
	AUC (CI 95%)	Cut-off	Sensitivity	Specificity	PPV	NPV	*p*-Value
MNBI at 5 cm	0.801 (0.674–0.929)	2937 Ω	76.9%	80.6%	58.8%	76.9%	<0.001
MNBI at 3 cm	0.802 (0.676–0.928)	2563 Ω	84.6%	80.6%	61.1%	93.55%	<0.001
PSPW index	0.87 (0.103–0.386)	64%	76.9%	88.9%	71.4%	91.43%	<0.001

## Data Availability

The data presented in this study are available on request from the corresponding author. The data are not publicly available due to privacy and ethical restrictions.
